# *Houttuynia cordata* Facilitates Metformin on Ameliorating Insulin Resistance Associated with Gut Microbiota Alteration in OLETF Rats

**DOI:** 10.3390/genes8100239

**Published:** 2017-09-22

**Authors:** Jing-Hua Wang, Shambhunath Bose, Soo-Kyoung Lim, AbuZar Ansari, Young-Won Chin, Han Seok Choi, Hojun Kim

**Affiliations:** 1 Department of Rehabilitation Medicine of Korean Medicine, Dongguk University, 814 Siksa, Goyang 10326, Gyeonggi-do, Korea; ewccwang@gmail.com (J.-H.W.); sklim1972@naver.com (S.-K.L.); abu.zar.0313@outlook.com (A.A.); 2 NosQuest, USPACE 1A-1103, Daewang Pangyoro 660, Bundang-gu, Seongnam-si 13494, Gyeonggi-do, Korea; shambose@yahoo.com; 3 College of Pharmacy, Dongguk University-Seoul, Dongguk-lo 32, Goyang 10326, Gyeonggi-do, Korea; f2744@dongguk.edu; 4 Department of endocrinology, Dongguk University, Dongguk-lo 32, Goyang 10326, Gyeonggi-do, Korea; hschoi402@dumc.or.kr

**Keywords:** *Houttuynia cordata*, insulin sensitivity, OLETF rat, gut microbiota, endotoxin

## Abstract

Metformin and *Houttuynia cordata* are representative anti-diabetic therapeutics in western and oriental medicine, respectively. The current study examined the synergistic anti-diabetic effect of *Houttuynia cordata* extraction (HCE) and metformin combination in Otsuka Long–Evans Tokushima Fatty (OLETF) rats. Fecal microbiota were analyzed by denaturing gradient gel electrophoresis (DGGE) and real-time PCR. Combining HCE + metformin resulted in significantly ameliorated glucose tolerance (oral glucose tolerance test (OGTT))—the same as metformin alone. Particularly, results of the insulin tolerance test (ITT) showed that combining HCE + metformin dramatically improved insulin sensitivity as compared to metformin treatment alone. Both fecal and serum endotoxin, as well as cytokines (tumor necrosis factor α (TNF-α) and interleukin 6 (IL-6)) were significantly ameliorated by HCE + metformin compared to metformin alone. Meanwhile, the activation of AMPK (adenosine monophosphate-activated protein kinase) by metformin was distinctly enhanced by HCE. Both of HCE and metformin evidently changed the gut microbiota composition, causing the alteration of bacterial metabolite, like short-chain fatty acids. *H. cordata*, together with metformin, exerts intensive sensibilization to insulin; the corresponding mechanisms are associated with alleviation of endotoxemia via regulation of gut microbiota, particularly *Roseburia*, *Akkermansia*, and Gram-negative bacterium.

## 1. Introduction

According to World Health Organization (WHO) epidemiological data, 9% of adults had diabetes worldwide in 2014 among which 90% belong to type 2 diabetes (T2D) [1,2]. T2D is a chronic systemic progressive metabolic disease characterized by hyperglycaemia due to insulin insufficiency and/or reduction of insulin sensitivity. Development of T2D generally involves three pathological manifestations, insulin resistance, postprandial hyperglycemia, and fasting hyperglycemia. Despite its discovery more than 60 years ago in Europe as a suppressor of hepatic glucose production, so far metformin is still considered as a first-line therapeutic for T2D [3]. However, in addition to diarrhea, nausea, cramps, vomiting, bloating, lactic acidosis, and abdominal pain, many other adverse effects of metformin are not a negligible issue in the clinic. Therefore, we attempted to explore an innovative agent which can act together with metformin for strengthening the therapeutic effectiveness and/or reducing the side effects.

*Houttuynia cordata* Thunb. (*H. cordata*) is a traditional edible and medicinal plant belonging to the Saururaceae family with widespread use in Asia. Similar to metformin in western medicine, *H. cordata* is derived from a natural source and has been frequently used as an anti-diabetic agent in oriental medicine. It has long been used as a tea in Japan, salad or juice in India, and a therapeutic agent in Korea and China for the treatment of various disorders including dysentery, fever, dyspepsia, hematochezia, and so on [4]. Accumulating evidence have indicated that an ample phenolic and flavonoid compounds-containing *H. cordata* exert hepatoprotective [5], anti-cancer [6], anti-obesity [7], anti-microbial [8], anti-viral [9], and anti-inflammatory [10] effects.

An increasing number of studies have suggested that commensal gut microbiota will become a promising therapeutic and predictive target for chronic metabolic disorders [11]. Ingestion of certain foods, including edible and medicinal herbs, evidently alters the composition and metabolites of gut microbiota, which results in avoidance of various diseases and occurrence of complications including T2D, obesity, and gastrointestinal disorders [12]. Therefore, modulation of gut microbiota by edible and medicinal herbs is regarded as a safe and feasible strategy for regulating metabolic homeostasis [13]. We have previously demonstrated that edible and medicinal herbs ameliorate high fat diet-induced obesity and endotoxemia via modulation of the gut microbiota and gut permeability in animal models [12,14,15]. Interestingly, earlier studies also have shown that both metformin and *H. cordata* have anti-diabetic activities which are closely associated with the regulation of gut microbiota [16]. Nevertheless, whether these two agents have the same or similar mechanisms of actions including alteration of gut microbiota or have synergistic anti-diabetic effects has not yet been elucidated.

The aim of this study was to evaluate the effect of *H. cordata* extract (HCE) and metformin in combination on diabetes in vitro as well in vivo using Otsuka Long-Evans Tokushima fatty (OLETF) rats, a genetic animal model of T2D with cholecystokinin (CCK)-1 receptor deficiency characterized by hyperphagia, hyperglycemia, hyperinsulinemia, obesity, and insulin resistance [17,18]. Moreover, we also attempted to find the potential mechanisms underlying the antidiabetic action of the above mentioned combination, particularly regarding the regulation of gut microbiota and their metabolites.

## 2. Materials and Methods

### 2.1. Preparation of *Houttuynia cordata* Extract

Korean Pharmacopoeia standards of *H. cordata* were purchased from Dongguk University Ilsan International Hospital (Goyang, Korea). After washing, 500 g of powdered *H. cordata* was extracted by ethanol (5 L) recycling reflux for 4 h at 70 °C. The extract was filtered through a qualitative filter paper (8 μm pore size, Whatman, Buckinghamshire, UK) and then vacuum lyophilized at −70 °C which eventually produced a yield of 29.1 g (5.82%). In addition, Metformin hydrochloride was obtained from Sigma-Aldrich (St. Louis, MO, USA).

### 2.2. Ultra-Performance Liquid Chromatography Based Fingerprinting

**Figure 1:**
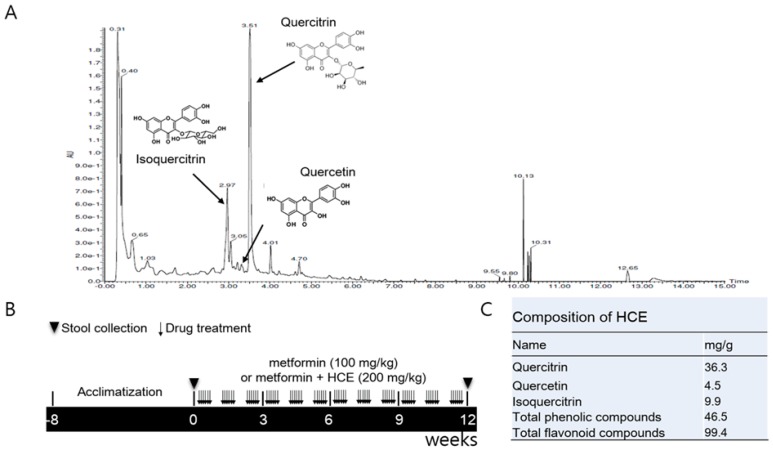
(**A**) Ultra-performance liquid chromatography (UPLC)-based fingerprinting of *Houttuynia cordata* extract (HCE) and in vivo experimental schedule. UPLC-based chromatographic analysis of HCE; (**B**) The chromatogram was produced at a wavelength of 205 nm. A synthetic scheme presents the experimental design; (**C**) The identification of the main compounds of HCE and determination of their concentrations were performed by comparison of their retention time and peak heights with those of the standards.

To find the major constituents of HCE, ultra-performance liquid chromatography (UPLC)-based fingerprinting was performed using a Waters Acquity^TM^ UPLC system (Waters, Milford, MA, USA) assembled with a binary pump, a 29 autosampler/injector, a column oven, and a UV/visible detector. Chromatographic separation of the extract and standards was achieved using an ACQUITY UPLC^®^BEH C18 column (2.1 mm × 150 mm, 1.7 μm), from Waters. The temperature of the samples and column was maintained at 10 and 30 °C, respectively and the injection volume was 4 μL. The mobile phase A was composed of 0.1% formic acid (*v*/*v*) in water and mobile phase B consisted of 0.1% formic acid (*v*/*v*) in acetonitrilie. Gradient for sample analysis was achieved by maintaining the proportion of mobile phase B at different time-points as follows: 10%, 0–1 min; 10–100%, 1–10 min; 100%, 10–12 min; 100–10%, 12–12.5 min; 10%, 12.5–15 min. The flow rate of mobile phase was maintained at 0.3 mL/min and the UV detection of eluate was carried out at a wavelength of 205 nm (Figure 1A). Quercitrin and isoquercitrin, the main components of HCE, were used as standards (Sigma-Aldrich) for the chromatographic analysis.

### 2.3. Determination of Total Phenolic and Flavonoid Contents of HCE

Total phenolic content of HCE was determined according to the Folin–Ciocalteu method [19] using gallic acid as a standard. Briefly, HCE samples were dissolved in 2% Na_2_CO_3_ and mixed with Folin–Ciocalteu’s reagent (Sigma-Aldrich) followed by incubation for 10 min at room temperature. The mixture was aliquoted into the 96-well plate and the absorbance was measured at 720 nm on a microplate reader (VersaMax, Molecular Devices, Sunnyvale, CA, USA). Total phenol content was determined using freshly prepared standard curve of gallic acid (Sigma-Aldrich). Total flavonoid content of HCE was measured according to the AlCl_3_ colorimetric method using catechin (Sigma-Aldrich) as a standard. Briefly, HCE was dissolved in 5% NaNO_2_ for 5 min and then mixed with 10% AlCl_3_ for 6 min. Following this, 1 M NaOH and distilled water were added and the absorbance of the resultant mixture was measured at 570 nm. Total flavonoid content was determined using freshly prepared standard curve of catechin (Figure 1C).

### 2.4. Animals and Experimental Schedule

Four-week old male LETO (Long–Evans Tokushima Otsuka, as control) and OLETF rats were purchased from Otsuka Pharmaceutical Co. (Tokushima, Japan). After 6 weeks of acclimatization, the OLETF rats were segregated into three groups. The metformin group was administered orally with metformin (100 mg/kg/day) for 12 weeks, while the metformin + HCE group was treated orally with a combination of metformin (100 mg/kg/day) and HCE (200 mg/kg/day) for 12 weeks. The OLETF and LETO groups were administered orally with distilled water for 12 weeks (Figure 1B). The animal study was approved by the Institutional Animal Care and Use Committee (IACUC-2014-037) of Dongguk University and performed in accordance with the Guide for the Care and Use of Laboratory Animals (Institute of Laboratory Animal Resources, Commission on Life Sciences, National Research Council, USA; National Academy Press: Washington, DC, USA, 1996).

### 2.5. Oral Glucose Tolerance Test and Intraperitoneal Insulin Tolerance Test

In each group, four animals were used for the oral glucose tolerance test (OGTT) and the remaining three were employed for the intraperitoneal insulin tolerance test (ipITT). For OGTT, three days prior the end of the animal experiment, 12 h fasting-adapted rats were treated with sterilized glucose solution (2 g/kg, Sigma-Aldrich) by oral gavage. Blood collected from the needle-punched tail vein was used as the sample. The blood glucose levels were measured at 0, 30, 60, 90, and 120 min post-glucose treatment using ACCU-CHEK Active (ACCU-CHECK, Mannheim, Germany). For ipITT, two days before the end of the animal experiment, biosynthetic human insulin (Eli Lilly and Company, Indianapolis, IN, USA) was administered to 12 h fasting-adapted rats by intraperitoneal injection (0.75 U/kg body weight). The glucose levels in the blood samples collected from the needle-punched tail vein at 0, 30, 60, 90, and 120 min post-insulin treatment were determined as mentioned above for the OGTT. The values of OGTT and ipITT were also corrected by baseline and expressed as baseline corrected areas under the curves (AUC) for evaluating the degree of impairment in glucose tolerance and insulin sensitivity, respectively.

### 2.6. Serum and Liver Biochemical Analyses

Liver and serum levels of triglyceride (TG), total cholesterol (TC), and high density lipoprotein (HDL) were determined using commercial enzymatic assay kits (Asan Pharmaceutical Co., Seoul, Korea) as per the kit manufacturer’s instructions. Serum insulin was measured using a rat insulin ELISA kit (Mercodia, Sweden). Briefly, 10 μL of samples or standards combined with 100 μL of enzyme conjugate solution were added to a pre-coated plate, followed by incubation for 2 h at room temperature. After washing 6 times with 700 μL wash buffer solution per well, 200 μL substrate 3,3′,5,5′-Tetramethylbenzidine (TMB) was added, followed by incubation for 15 min at room temperature. Finally, 50 μL stop solution was added and the plate was read immediately on a microplate reader (Molecular Devices, Sunnyvale, CA, USA) at 450 nm. The concentration of fasting serum insulin was calculated using a freshly prepared insulin standard curve.

### 2.7. Analysis of Serum and Fecal Endotoxin Levels

Serum and fecal endotoxin levels were determined using a Limulus amebocyte lysate (LAL) kit (ENDOSAFE, Charleston, SC, USA) according to the kit manufacturer’s protocol. Briefly, serum or prepared fecal supernatant samples were added to 5 EU/mL standard spiked wells. Following addition of 100 μL of LAL reagent, the kinetic absorbance of the mixture was measured at 405 nm and the reaction onset times of the samples were compared to the standard curve.

### 2.8. Measurement of Serum Pro-Inflammatory Cytokines

The serum levels of tumor necrosis factor α (TNF-α), interleukin 6 (IL-6), and IL-1β were determined using commercial ELISA kits following the instructions of the kit manufacturers (BioLegend, San Diego, CA, USA for TNF-α and IL-6; and BD Biosciences, San Jose, CA, USA for IL-1β).

### 2.9. Histopathological Analysis

Paraffin-embedded liver and pancreas tissues were sectioned into 6-μm thickness using microtome (Leica RM2235, Leica, Nussloch, Germany) and the sections were fixed on silicon-coated glass slides. Liver sections were stained with hematoxylin (Merck, Boston, MA, USA) and counter-stained with eosin (Sigma-Aldrich). Pancreatic sections were stained with aldehyde-fuchsin (Sigma-Aldrich) followed by hematoxylin staining. The stained tissue sections were examined at 200× magnification under a light microscope Olympus BX61 (Olympus, Tokyo, Japan) and photographed (100 μm) using a digital camera Olympus DP70 (Olympus). The size and area of pancreatic islets were measured using Image J, an open source Java image processing program developed by the National Institutes of Health (NIH, Bethesda, Rockville, MD, USA).

### 2.10. Quantification of Fecal Short Chain Fatty Acid

Stored rat feces were added to HPLC-grade water (Sigma-Aldrich) and sonicated to prepare tissue homogenate. Following centrifugation of the homogenate at 13,000× *g* for 3 min, the supernatant was filtered through a 0.2 μm filter (Millipore, Bedford, MA, USA) Prepared samples were analyzed using an Agilent 1220 Infinity LC system (Agilent, Santa Clara, CA, USA) equipped with an Aminex-87H column (150 mm, 4.6 mm, Bio-Rad, Hercules, CA, USA) and a diode array detector. Pure acetic acid, propionic acid, and butyric acid (Sigma-Aldrich) were used as standards. Samples were eluted with 0.008 N sulfuric acid for 30 min at a flow rate of 0.6 mL/min. All chromatograms were obtained using a wavelength of 215 nm and data acquisition and calculation was performed using Agilent chemstation software (Agilent).

### 2.11. Western Blot Analysis

The frozen liver tissues of three animals from each group were homogenized and mixed together in radioimmunoprecipitation assay (RIPA) buffer containing protease and phosphatase inhibitor (Abcam, Cambridge, MA, USA). Following centrifugation of the homogenate, the supernatant was isolated and its total protein content was determined using a bicinchoninic acid (BCA) kit (Thermo Scientific, Rockford, lL, USA). After denaturation, the proteins were separated in 10% Sodium Dodecyl Sulphate-Polyacrylamide Gel Electrophoresis (SDS-PAGE) gel and then transferred to a polyvinylidene fluoride (PVDF) membrane (GE Healthcare Life Science, Freiburg, Germany) using a Mini-PROTEAN Tetra Cell system (Bio-Rad). The membranes were blocked with 5% skim milk (Sigma-Aldrich) in TBST (Tris-buffered saline containing 0.1% Tween 20, *v*/*v*) for 1 h followed by treatment with primary antibodies (Table S3) (1:10,000) overnight at 4 °C. The membranes were washed thoroughly and then incubated with appropriate horseradish peroxidase-conjugated secondary antibodies for 1 h. Finally, the membranes were treated with SUPEX ECL solution (Neuronex, Pohang, Korea) and the immunoreactive protein bands were detected and photographed using FUJIFILM LAS3000 (Fujifilm, Tokyo, Japan).

### 2.12. Fecal Microbial Analysis Using Denaturing Gradient Gel Electrophoresis and Real-Time PCR

Fresh rat stools were collected separately at the beginning and the end of the treatment schedule. The fecal microbial genomic DNA was isolated using a QIAamp DNA Stool Mini Kit (Qiagen, Valencia, CA, USA) following the kit manufacturer’s instructions. The purity and concentration of the prepared DNA samples were checked using a NanoDrop™ (Thermo Scientific), and the quality of DNA was further verified by gel-electrophoresis. The extracted DNA was subjected to routine PCR amplification using universal primers for the bacterial 16S rRNA gene (5′-AGA GTT TGA TCC TGG CTC AG-3′ and 5′-AAG GAG GTG ATC CAG CC-3′). The first PCR products were electrophoresed on 1% agarose gel and then stained with ethidium bromide (EB, Bio-Rad). Only 1.5 kb DNA bands on gels were isolated following which DNA was extracted using an Accuprep gel purification kit (Bioneer, Daejeon, Korea). The second round of PCR involved the amplification of the V3 region of 16S rDNA using the primer with a GC-clamp (314f-GC: 5′-CGC CCG CCG CGC GCG GCG GGC GGG GCG GGG GCA CGG GGG GCC TAC GGG AGG CAG CAG-3′ and 518r: 5′-ATT ACC GCG GCT GCT GG-3′). Denaturing gradient gel electrophoresis (DGGE) of the second PCR products was carried out using the DCode universal mutation detection system (Bio-Rad). Briefly, the PCR products were electrophoresed in 40% acrylamide/bis (37.5:1) gels with denaturing gradients from 30 to 60% (where 100% is 7 M urea and 40% (*v*/*v*) formamide) in Tris-acetate-EDTA (TAE) buffer (ViroMed, Seoul, Korea) at 80 V for 15 h. The gels were stained with EB and subsequently were photographed under FUJIFILM LAS3000 (Fujifilm). All data were analyzed using an unweighted pair group method with an arithmetic means (UPGMA) clustering procedure based on genetic similarity expressed by the Jaccard coefficient. Principal component analysis (PCA) was also performed on the DGGE profiles using Bionumerics software (version 3.0, Applied Maths, Saint-Martens-Latem, Belgium).

Real-time PCR was performed on a LightCycler 480 system (Roche Applied Science, Indianapolis, ID, USA) using a lightCycler FastStart DNA Master SYBR Green kit (Roche Applied Science). The primer sequences used for targeting the 16S rRNA gene of the bacteria are listed in Table S2. The standard conditions for the PCR amplification reactions were followed as described previously [14]. The quantification of bacterial relative abundance was performed by 2^−Ct^ calculations. The final results are expressed as normalized fold values relative to the LETO group.

### 2.13. Statistical Analysis

All data were analyzed by one-way ANOVA followed by least significant difference (LSD) post hoc test (SPSS 17.0, Chicago, IL, USA), and the results were expressed as the mean ± standard deviation (SD). *p* < 0.05 was regarded as statistically significant.

## 3. Results

### 3.1. Composition of *Houttuynia cordata* Extract

Total phenolic and flavonoid compounds of HCE were found to be 46.5 mg/g of gallic acid and 99.4 mg/g of rutin equivalent, respectively. Further analyses have revealed that HCE contains 36.3 mg/g of quercitrin, 4.5 mg/g of quercetin, and 9.9 mg/g isoquercitrin (Figure 1C).

### 3.2. Amelioration of Insulin and Glucose-Related Parameters

**Figure 2:**
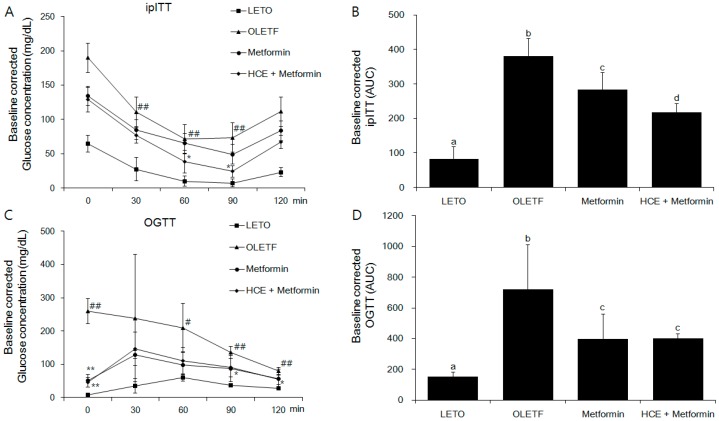
Impact of metformin either alone or in combination with HCE on the insulin sensitivity and glucose tolerance in OLETF rats. (**A**) Intraperitoneal insulin tolerance test (ipITT) and (**C**) oral glucose tolerance test (OGTT) were conducted on the animals at the last week of treatment schedule and the corresponding baseline corrected areas under the curves (AUCs) (**B**,**D** respectively) were constructed as described in the Materials and Methods section. # *p* < 0.05, ## *p* < 0.01 compared to LETO group; * *p* < 0.05, ** *p* < 0.01 compared to the OLETF group, as analyzed by one way ANOVA test. Data in bar diagrams with different letters are significantly different (*p* < 0.05) according to one way ANOVA analysis.

**Figure 3:**
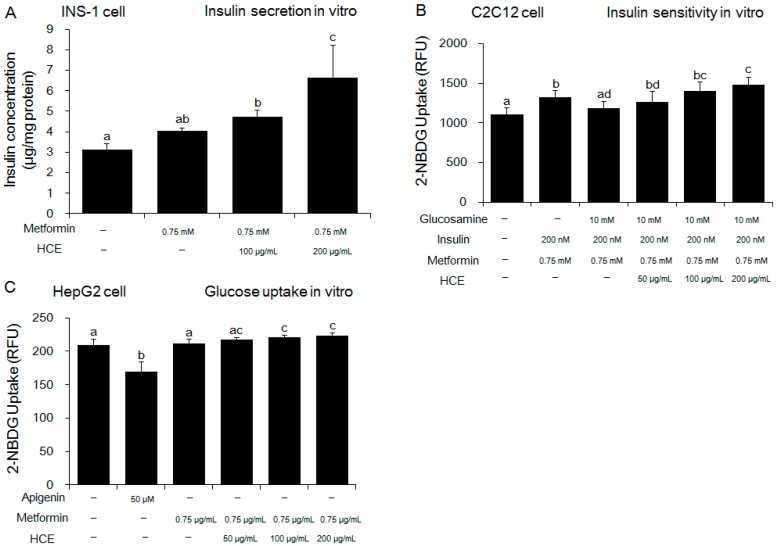
Effect of metformin either alone or in combination with HCE on the in vitro insulin secretion, insulin sensitivity, and glucose uptake. (**A**) Insulin secretion, (**B**) insulin sensitivity and (**C**) glucose uptake were measured using INS-1 (an insulin-secreting cell line), C2C12 (an immortalized mouse myoblast cell line), and HepG2 (a human liver cancer cell line) cells, respectively as mentioned in the Materials and Methods section. Data with different letters are significantly different (*p* < 0.05) according to one way ANOVA analysis.

Exposure of OLEFT rats to either metformin alone or metformin + HCE did not produce any noticeable effect on the body weight gain (Figure S1A). However, in an ipITT test, treatment with metformin + HCE led to significant inhibition of the glucose level at 60 and 90 min (Figure 2A), suggesting improvement of insulin resistance, while in the OGTT test, treatment with metformin + HCE significantly reduced the glucose level at 0, 90, 120 min (Figure 2C). Treatment with both metformin and metformin + HCE significantly reduced the fasting blood glucose and serum insulin levels (Figure S1B,C) as well as Homeostatic Model Assessment for Insulin Resistance (HOMA-IR, Figure S1D), baseline corrected ipITT glucose area under the curve (AUC, Figure 2B), and baseline corrected OGTT glucose AUC (Figure 2D). Furthermore, baseline corrected ipITT glucose AUC in the metformin + HCE group was found to be significantly lower than that in metformin group. In alignment, co-exposure to HCE caused marked enhancement in the insulin secretion, insulin sensitivity, and glucose uptake in a concentration-dependent manner in metformin-treated INS-1 (an insulin-secreting cell line, at high glucose level), C2C12 (glucosamine-induced), and HepG2 cells, respectively (Figure 3A–C).

### 3.3. Improvement of Serum Lipid Profile and Hepatic Triglycerides

Serum levels of TG and TC, and TG/HDL ratio were significantly higher and HDL level was significantly lower in the OLEFT group compared to the LETO group (Table 1). Meanwhile, hepatic TG was markedly increased in the OLEFT group compared to LETO group. Treatment of OLEFT rats with metformin + HCE, but not metformin alone, resulted in a significant increase in the serum HDL level and significant decreases in the serum TG and TC levels as well as TG/HDL ratio (Table 1). However, both of OLEFT rats treated with metformin alone and metformin + HCE remarkably reduced TG concentration in liver tissue compared to OLETF group.

### 3.4. Suppression of Systemic Endotoxin and Inflammation

**Figure 4:**
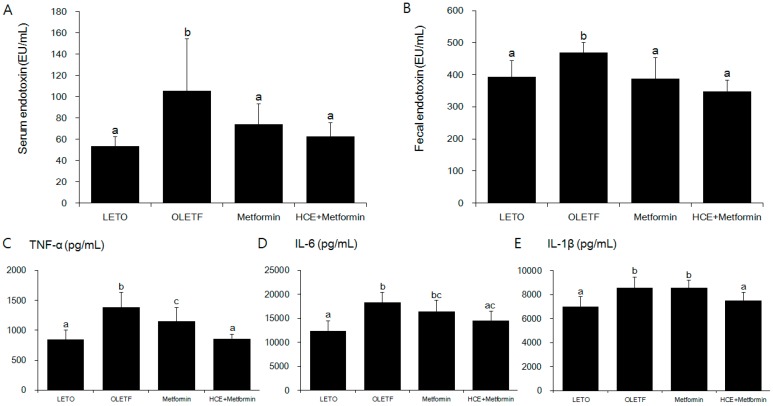
Effect of metformin either alone or in combination with HCE on the serum and fecal endotoxin levels (**A**,**B** respectively); and the vital serum proinflammatory cytokines (**C**–**E**) in OLETF rats. Data with different letters are significantly different (*p* < 0.05) according to one way ANOVA analysis. IL-6, interleukin 6; TNF-α, tumor necrosis factor alfa.

As expected, serum and fecal endotoxin levels were significantly higher in the OLETF group compared to the LETO group (Figure 4A,B). Treatment of OLETF rats with metformin significantly decreased the fecal endotoxin content, but did not cause any significant change in the serum endotoxin level, while both the serum and fecal endotoxin levels in the metformin + HCE group were found to be significantly lower than those in either the untreated or metformin-treated OLETF rats (Figure 4A,B). The serum levels of inflammatory cytokines TNF-α, IL-6 and IL-1β were significantly higher in the OLETF group compared to the LETO group (Figure 4C–E). Treatment of OLETF rats with metformin significantly attenuated the serum TNF-α level, but did not cause any significant change in the serum IL-6 and IL-1β concentrations. In contrast, the serum levels of all the above-mentioned cytokines were significantly lower in the metformin + HCE group compared to the OLETF group. Additionally, the serum levels of TNF-α and IL-1β in the metformin + HCE group were found to be significantly lower compared to the metformin group.

### 3.5. Activation of adenosine monophosphate-activated protein kinase

**Figure 5:**
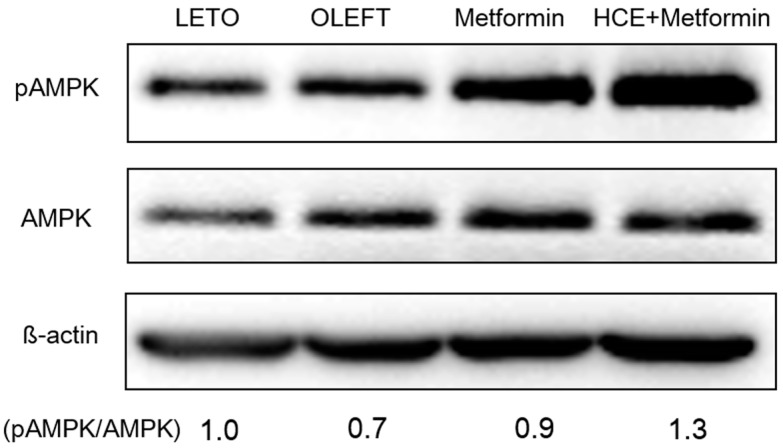
Impact of metformin either alone or in combination with HCE on the activation of hepatic adenosine monophosphate-activated protein kinase (AMPK) in OLETF rats as estimated by Western blotting. The density of the phospho-AMPK bands in the blot was quantified by densitometric analysis and normalized to the amount of non-phospho form of AMPK.

Treatment of OLETF rats with metformin triggers the phosphorylation of hepatic AMPKα at Thr^172^ position resulting in the activation of AMPK. Exposure of metformin-treated rats to HCE resulted in a marked enhancement in AMPK phosphorylation (Figure 5).

### 3.6. Histopathological Analysis

**Figure 6:**
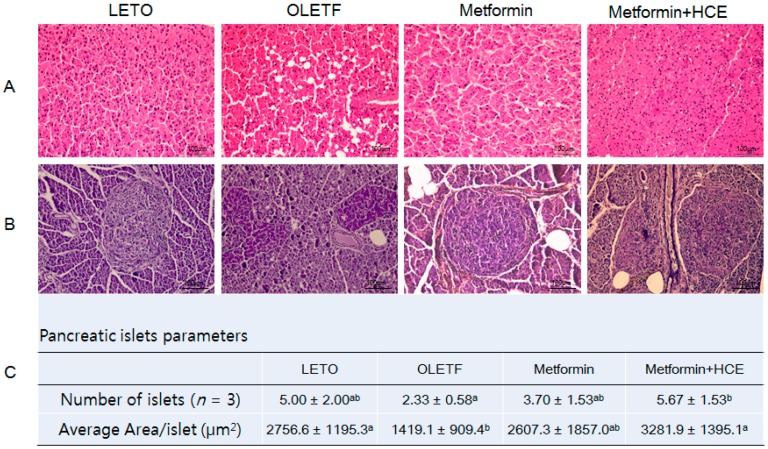
Effect of metformin either alone or in combination with HCE on the histological architecture of the liver and pancreas, and the abundance of pancreatic islets in OLETF rats. At the termination of the experimental period, the liver (**A**) and pancreas (**B**) were excised rapidly following which tissue sections were prepared and stained with hematoxylin. The histological examination of the tissue sections was performed under a light microscope (200× magnification). Number of pancreatic islets and average area/islet are shown (**C**). Data with different letters are significantly different (*p* < 0.05) according to one way ANOVA analysis.

Histophathological evaluation of the liver of OLETF rats revealed an abnormal architecture of the hepatic tissue characterized by marked accumulation of lipid as vacuoles (Figure 6A). Treatment of OLETF rats with metformin caused a marked amelioration in these pathological conditions and such hepatoprotective activity of metformin was further improved upon co-treatment of animals with HCE. Additionally, the number and area of islets of Langerhans in the pancreatic tissues of the OLETF group were noticeably lower compared to the LETO group (Figure 6B). An exposure of OLETF rats to metformin + HCE, but not metformin alone, resulted in a significant increase in both the number and size of islets (Figure 6C).

### 3.7. Modification of Gut Microbiota Distribution and Profile of SCFAs

**Figure 7:**
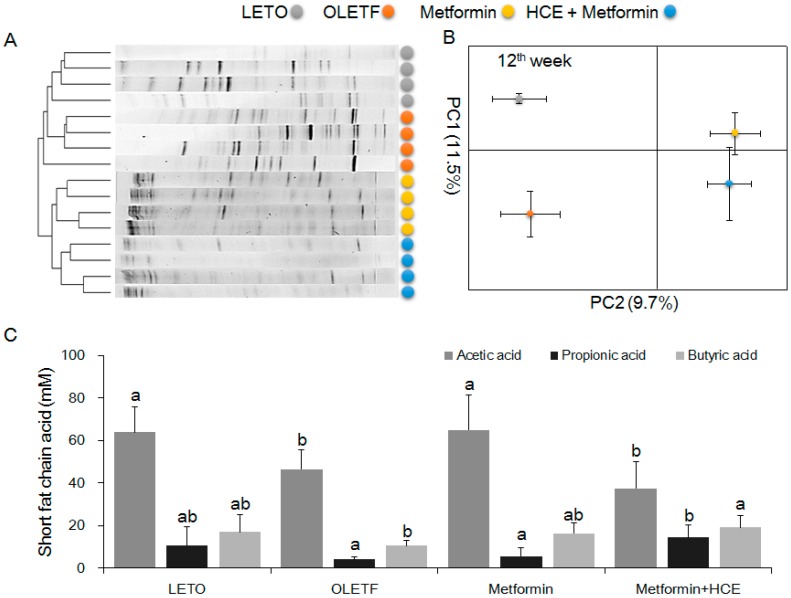
Impact of metformin either alone or in combination with HCE on the gut microbial population and the abundance of short chain fatty acids in the stool of OLETF rats. (**A**) The microbial communities in the fecal samples were analyzed by PCR-denaturing gradient gel electrophoresis (DGGE) as described in the Materials and Methods section; (**B**) Principal component analysis (PCA) of the data was performed based on distance matrix (two-dimensional array) for the evaluation of similarity between bacterial communities; (**C**) The levels of fecal acetic acid, propionic acid, and butyric acid are shown. Data with different letters are significantly different (*p* < 0.05) according to one way ANOVA analysis.

**Figure 8:**
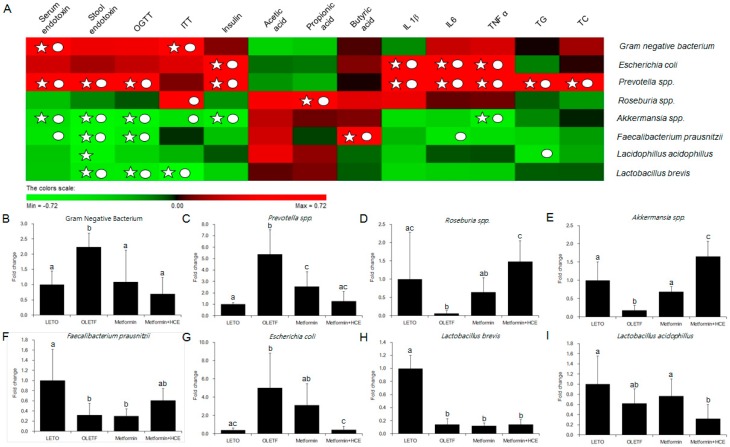
Relative abundance of gut microbiota and correlation between gut microbial relative abundance and host parameters. (**A**) The correlation analysis was performed using two-tailed Pearson’s correlation test and diagramed by PermuMatrix software (version 1.9.3 EN) using heatmap plots; (**B**–**I**) *R* value greater than 0.4 indicates a positive correlation (red color), while *r* value less than −0.4 indicates a negative correlation (green color). A symbol of ○ indicates value of Pearson *r* > 0.4 or *r* < −0.4 and a symbol of ☆ indicates statistical significance of *p* value less than 0.05. The relative abundance of the 16S rRNA gene of the gut bacteria in stool samples was determined using real-time PCR as described in the Materials and Methods section. The results are expressed as normalized fold values relative to the normal group. Data are expressed as the mean ± SD (*n* = 7). Data with different letters are significantly different (*p* < 0.05) according to one way ANOVA analysis.

The stool samples of the animals demonstrated a noticeable difference in the distribution of gut microbial community between the LETO and OLETF groups as reflected by the distinct clustering and PCA patterns of these two groups in the DGGE profile (Figure 7A,B). Treatment of OLETF rats with both metformin and metformin + HCE resulted in a noticeable altered gut microbial distribution pattern (Figure 7A,B). A significantly higher population of the entire Gram-negative bacteria as well as *Prevotella* spp. and *Escherichia coli* were seen in the OLETF group compared to the LETO (Figure 8). However, treatment of OLETF rats with either metformin or metformin + HCE resulted in a significant decrease in the abundance of Gram-negative bacteria and *Prevotella* spp., more pronouncedly in the case of later treatment. While exposure of OLETF rats to metformin + HCE, but not metformin alone, significantly reduced the gut population of *E. coli*. A significantly lower population of *Roseburia* spp., *Akkermansia* spp., *Faecalibacterium prausnitzii*, and *Lactobacillus brevis* were seen in the OLETF group compared to the LETO. Nevertheless, an exposure of OLETF rats to metformin + HCE, but not metformin alone, significantly increased the population of *Roseburia* spp., while treatment of OLETF rats with both metformin and metformin + HCE resulted in a significant increase in the abundance of *Akkermansia* spp., more pronouncedly in the case of later treatment. The fecal acetic acid level was found to be significantly lower in the OLETF group compared to the LETO (Figure 7C), while treatment of OLETF rats with metformin, but not metformin + HCE, significantly increased the fecal acetic acid level. Insignificant but noticeable lower levels of fecal propionic and butyric acids were seen in the OLETF group compared to the LETO (Figure 7C). However, treatment of OLETF rats with metformin + HCE, but not metformin alone, significantly augmented the fecal propionic and butyric acid levels.

## 4. Discussion

Medicinal plants are traditionally used as an important source in the search for suitable active principle(s) against a variety of therapeutic areas and are continuously evaluated for their possible potential pharmacological activities in the regulation of adverse conditions including elevated blood glucose level in diabetes. Emerging evidence supports the anti-hyperglycemic activity of *H. cordata* [20,21]. Furthermore, previous studies revealed that like metformin, *H. cordata* ameliorated T2D through restoration of insulin sensitivity, lowering blood glucose, and improving lipid metabolism [22,23]. Additionally, both animal and clinical studies have shown that certain herbal formulas can potentiate the effect of metformin on lipid and glucose metabolism in diabetic state [24,25,26]. These all are in keeping with our findings in in vitro studies where combination of HCE and metformin treatment demonstrated amelioration of hepatic glucose uptake, insulin secretion, and insulin sensitivity in a concentration-dependent manner, more pronouncedly than metformin treatment alone. To further confirm these synergistic anti-diabetic actions and understand the underlying mechanisms in vivo, OLETF rats were used a genetically diabetic animal model in our study. The results showed that treatment with metformin + HCE was more beneficial than metformin alone in the improvement of glucose metabolism and insulin sensitivity.

Dyslipidemia, a frequent complication of T2D, is characterized by low levels of HDL and high levels of TG [27,28], while the TG/HDL ratio is also considered to be a potential marker for insulin resistance in the clinic [29]. A high ratio is mostly associated with incidence of insulin resistance and T2D [30,31]. The properties of metformin in lowering serum TG and TC levels as well as increasing HDL level were previously reported [32]. In our study, similar kinds of effects of this drug were also observed in OLETF rats, although the changes in the serum lipid parameters were found to be insignificant. However, co-exposure of metformin-treated rats to HCE resulted in a significant increase in the serum HDL level and significant reductions in the serum TG and TC levels as well as TG/HDL ratio, suggesting that the beneficial effects of metformin on serum lipid parameters are potentiated by HCE. This is in agreement with a recent report showing the anti-hyperlipidemic effect of *H. cordata* extract which significantly reduced the cholesterol and TG levels in streptozotocin-induced diabetic rats [21]; an earlier study demonstrated the anti-obesity effect of water extract of *H. cordata* leaves (WEH) in mice with high-fat-diet-induced obesity [7]. This study further showed that WEH inhibited the corn oil-induced increase in plasma triglyceride levels and prevented fat absorption in mice. WEH also suppressed the oleic acid- and glycerol-induced increase in the levels of plasma non-esterified fatty acids (NEFA) and glycerol, respectively. The authors postulated that WEH inhibited fat absorption in the small intestine through two mechanisms: (1) suppression of the enzymatic activity of digestive lipases which break down TG to NEFA and monoglycerides or glycerol, and (2) reduction in the uptake of these broken products by the intestinal epithelial cells.

In keeping with other chronic metabolic disorders, T2D is also characterized as a chronic low-grade inflammation disease [33,34], and metabolic endotoxin-provoked inflammation is closely associated with insulin resistance and dysglycemia [35]. Thus, low-grade inflammation is deemed to play a pivotal role in insulin resistance and diabetes. This is also in agreement with our study where serum and fecal endotoxin levels were found to be significantly higher in the OLETF group compared to the LETO group. Endotoxin is known to trigger synthesis of inflammatory cytokines such as interferon-γ, TNF-α, IL-6 and IL-1β in macrophages [36,37,38]. According to previous reports, metformin can effectively prevent several endotoxin-induced diseases [39,40], while anti-inflammatory action is a common property of both metformin and *Houttuynia cordata* [10,41]. In our study, the levels of both serum and fecal endotoxin as well as serum TNF-α and IL-1β concentrations in the metformin + HCE group were significantly lower than those in the metformin group, indicating that the anti-inflammatory activity of *Houttuynia cordata* is stronger than that of metformin. It was reported that *Houttuynia cordata* supercritical extract (HSE) was able to reduce the production of TNF-α and prostaglandin E2 (PGE2) in a carrageenan-air pouch model of inflammation in mice, suggesting that HSE exerts anti-inflammatory effects by inhibiting TNF-α and cyclooxygenase 2 (COX-2)-PGE2 pathways [42]. Using lipopolysaccharide (LPS)-stimulated RAW 264.7 macrophages as an in vitro inflammatory model, it was shown that ethyl acetate fraction of *Houttuynia cordata* extract (HCE-EA) downregulated nitric oxide (NO), PGE2, TNF-α, and IL-6 production, as well as inducible nitric oxide synthase (iNOS) and COX-2 expression [43]. Moreover, HCE-EA suppressed nuclear translocation of the nuclear factor kappa-light-chain-enhancer of activated B cells (NF-κB) p65 subunit which correlated with an inhibitory impact on the nuclear factor of the kappa light polypeptide gene enhancer in B-cells inhibitor, alpha (IκBα) phosphorylation. HCE-EA also inhibited the activation of mitogen-activated protein kinases (MAPKs) [43]. Taking everything into consideration, it was suggested that the anti-inflammatory activities of HCE-EA may be exerted through the inhibition of pro-inflammatory mediators via the suppression of NF-κB and MAPK signaling pathways [43]. Based on our results, it is conceivable that the anti-inflammatory property of metformin is potentiated by HCE which might account for the improvement in insulin sensitivity in metformin-treated OLETF rats in response to the exposure to HCE.

The gut microbiota modulates the biochemical activity of the host by providing extra metabolic functions and regulating a variety of molecular events of cellular differentiation and gene expression through host–microbe interactions [44]. Disturbance in the balance between beneficial and detrimental gut microbiota has been suggested to promote intestinal inflammation-induced metabolic disorders (insulin resistance, diabetes, and obesity) [35,45]. Based on these and accumulating evidence [46], gut microbiota intervention is regarded as a feasible therapeutic strategy for ameliorating the above mentioned diseases. Interestingly, it has been shown that metformin exerts antidiabetic effects via alteration in the intestinal bacterial composition, function, and metabolites in both human and animals [16,47]. Metformin also demonstrated reverting effects on the high-fat diet-induced structural changes of gut microbiota [48]. Our previous findings revealed that natural products improve chronic metabolic diseases and related endotoxemia through modulation of the distribution of the gut microbiota [12,14]). Although no direct study fully evaluated the relationship between *H. cordata* and gut microbiota in vivo, the antibiotic activities of *H. cordata* have already been validated in vitro [8]. Therefore, modulation of the gut microbiota and its metabolites is another conceivable mechanism of the synergistic action of metformin and *H. cordata*.

In the current study, the profile of gut microbial population in OLETF rats was noticeably altered upon treatment with either metformin alone or metformin + HCE. Despite a moderate difference in the entire gut microbial profile was seen between the metformin and metformin + HCE group, detailed gut bacterial data revealed that in OLETF rats, metformin + HCE treatment more markedly suppressed the Gram-negative bacteria compared to the treatment with metformin alone. These data are also consistent with our results on the endotoxin and pro-inflammatory cytokine profiles. Particularly, at the genus level, several proven diabetic-related microbial genus [49], including *Prevotella*, *Roseburia,* and *Akkermansia* were more distinctly altered by the metformin + HCE treatment compared to the metformin treatment alone, in a positive direction according to the previous evidence [16,50,51]. It has been revealed that an increase in the *Akkermansia* spp. population induced by metformin treatment improves glucose homeostasis in diet-induced obese mice [16]. In an earlier study, based on the experimental evidence, it has been hypothesized that metformin shifts gut microbiota composition through the enrichment of *Akkermansia muciniphila* [52], the mucin-degrading bacterium which has also been shown to be inversely correlated with the onset of inflammation, altered adipose tissue metabolism and metabolic disorders during obesity in mice [53]. However, in the clinical scenario, subjects with higher gene richness and *A. muciniphila* abundance exhibited the healthiest metabolic status, particularly in fasting plasma glucose, plasma triglycerides and body fat distribution [54]. Accumulating evidence also suggests that T2D is associated with a decrease in genera including *Roseburia* spp. [47,55]. Increased levels of *Roseburia* were found to be associated with improved insulin sensitivity following gut microbiota transplantations from lean donors to recipients with metabolic syndrome [56]. In a clinical study, *Prevotella* was significantly lower in the children with diabetes compared with the healthy children [57]. It has been shown that *Prevotella copri* can induce insulin resistance and aggravate glucose intolerance in mice [58]. Additionally, our results revealed that an exposure of OLETF rats to metformin + HCE, but not metformin alone, significantly decreased the gut population of *E. coli*. It has been observed that survivors with E. coli O157:H7-induced diarrhea-associated overt hemolytic-uremic syndrome have a significantly increased incidence of diabetes due to complete insulin deficiency, which may recur several years after the initial infection [59]. Furthermore, in our study based on the interrelation of gut microbiota and host parameters (Figure 8A), Gram-negative bacteria showed a significant positive correlation with the serum endotoxin and insulin tolerance; *Prevotella* exhibited a positive correlation with the serum and stool endotoxin, glucose tolerance, serum insulin, inflammatory cytokines and total cholesterol, whereas *Akkermansia* showed a negative correlation with serum and stool endotoxin, glucose tolerance and serum insulin. These findings are also in accordance with previous results [60,61]. Taken together, it is conceivable that *H. cordata* improves the pharmacological action of metformin in modulating the gut microbiota toward a more beneficial direction leading to the improvement of insulin sensitivity.

Short chain fatty acids (SCFAs), the members of specific short chain fatty acids, are generated by gut microbiota-mediated fermentation of carbohydrates [62]. These molecules play a vital role in the improvement of glucose homeostasis and insulin sensitivity and protection against the development of obesity and insulin resistance [63,64,65]. Therefore, an alteration in the gut microbial population can directly influence the enteral SCFA concentration, leading to the changes in glucose and lipid metabolism [66]. In the current study, HCE and metformin synergistically elevated fecal levels of butyric and propionic acids in the OLETF rats, where modulation of gut microbial modification by these two agents may play a vital role.

AMPK, an energy sensor, plays a vital role in glucose and lipid metabolism [67]. Upon activation, AMPK triggers glucose uptake and lipid oxidation to generate energy, while turning off energy-consuming processes including glucose and lipid synthesis to maintain energy balance [67]. AMPK regulates whole-body glucose homeostasis by modulating metabolism in multiple peripheral tissues including liver [67]. The anti-diabetic action of metformin and anti-lipidemic activity of *H. cordata* have been shown to be mediated through the AMPK-dependent pathway [68,69]. In our study, HCE markedly facilitated the metformin-mediated activation of AMPK, suggesting the beneficial impact of HCE on metformin in maintaining glucose and lipid homeostasis.

Flavonoids, a diverse group of natural polyphenol compounds, are well known for their beneficial effects on health. In the present study, *H. cordata* exhibited high flavonoid and phenolic contents including quercitrin, isoquercitrin, and quercetin. Quercetin is known to cause increase of hepatic glucokinase activity, augmentation of liver glucose uptake, decrease of hepatic glycogenolysis and gluconeogenesis, reduction of blood glucose, enhancement in serum insulin concentration, improvement in insulin sensitivity and glucose tolerance, and reduction of plasma cholesterol and triglyceride levels in diabetic animal models [70]. Both quercetin and its derivatives are known to activate AMPK and stimulate glucose uptake in muscle cells [70]. Accumulating evidence indicates that glycosides, including rutin and quercitrin can be converted to quercetin by the enzymatic action of gut microbial glucosidase and rhamnosidase [71]. Taking everything into consideration, it is conceivable that an alteration in gut microbial community by *H. cordata* might be one of the potential reasons for its anti-diabetic action which is mediated through bioconversion of the herbal components.

Finally, it is realized that the small sample size and effective evaluation using only a relatively rare genetic diabetic animal model are limitations of this study and therefore, the results may be difficult to extrapolate to humans straightforwardly. The gut microbial alteration might not be the exclusive mechanism for ameliorating insulin resistance. A previous study has shown that the antidiabetic effect of *H. cordata* is attributed to an upregulation of GLUT-4 and potential antioxidant activity [20]. In the future, a carefully controlled clinical trial needs to be performed for confirmation of the anti-diabetic action of *H. cordata* and additional investigations using an antibiotics-treated or germ-free animal model to explore the microbiological mechanism(s).

## 5. Conclusions

In conclusion, exposure to metformin in combination with *H. cordata* extract being enriched with high flavonoid and phenolic ingredients improved insulin sensitivity and hyperlipidemia which was associated with the reduction of endotoxin and inflammatory stress. The most probable underlying mechanism was found to be the regulation of gut microbiota, particularly Gram-negative bacteria, *Roseburia*, and *Akkermansia*. Thus, the combination of *H. cordata* and metformin can be a more efficient candidate for the treatment of patients suffering from metabolic syndrome, particularly T2D and hyperlipidemia.

## Figures and Tables

**Table 1:** Serum lipid parameters of the different experimental groups.

Groups	LETO	OLETF	Metformin	Metformin + HCE
TG (mg/dL)	35.2 ± 17.8 ^a^	118.0 ± 32.7 ^b^	113.9 ± 41.4 ^bc^	79.5 ± 35.3 ^c^
TC (mg/dL)	122.5 ± 15.1 ^a^	169.9 ± 21.2 ^b^	150.6 ± 40.4 ^ab^	132.8 ± 31.1 ^a^
HTG (mg/g tissue)	50.3 ± 6.0 ^a^	98.6 ± 13.8 ^b^	75.2 ± 5.3 ^c^	79.4 ± 6.5 ^c^
HDL (mg/dL)	16.1 ± 1.7 ^a^	12.2 ± 1.6 ^b^	14.9 ± 2.8 ^ab^	15.6 ± 3.5 ^a^
TG/HDL ratio	2.2 ± 1.1 ^a^	9.7 ± 2.7 ^b^	8.5 ± 3.9 ^ab^	5.8 ± 3.3 ^a^

Abbreviations: TC: total cholesterol; HDL: high density lipoprotein; TG: triglyceride; HTG: hepatic triglyceride; LETO: Long–Evans Tokushima Otsuka; OLETF: Otsuka Long–Evans Tokushima Fatty; HCE: Houttuynia cordata extract. Data were expressed as mean ± standard deviation (SD), different letters indicate significantly different according to one way ANOVA analysis followed by LSD post-hoc test (*p* < 0.05, *n* = 7).

## Supplementary Materials

The following are available online at www.mdpi.com/2073-4425/8/10/239/s1. Supplementary Methods and Materials: S1: Animal experimental schedule; S2: Cell culture; S3: Determination of insulin secretion and sensitivity, and glucose uptake in vitro. Figure S1: Profile of body mass and glucose metabolism, Table S1: Body weight, fat and organ weights, food intake and food efficiency ratio, and other parameters of the different experimental groups, Table S2: List of primers used for the gut microbial analysis by real-time PCR, Table S3: List of antibodies used for western blot analysis.
