# Supplementary material for: Houttuynia cordata Facilitates Metformin on Ameliorating Insulin Resistance Associated with Gut Microbiota Alteration in OLETF Rats
Source: Genes (Basel). 2017 Sep 22;8(10):239. doi: 10.3390/genes8100239 (PMC5664089; doi:10.3390/genes8100239)
Supplement: Supplementary file 1 [file genes-08-00239-s001.pdf]

genes

Supplementary Materials: *Houttuynia cordata* Facilitates Metformin on Ameliorating Insulin Resistance Associated with Gut Microbiota Alteration in OLETF Rats

Jing-Hua Wang, Shambhunath Bose, Soo-Kyoung Lim, AbuZar Ansari, Young-Won Chin, Han Seok Choi and Hojun Kim

Supplementary methods and materials

S1 Animal experimental schedule

Animal body weight and food-intake was measured once a week for the entire experimental period. The food efficiency ratio (FER) was calculated as the total average body weight gain divided by the total average food intake per day. Fresh stool samples were collected the day before sacrifice and then stored at -80℃ until analyzed. After 12 weeks of dosing followed by 12 h of fasting, all animals were sacrificed under anesthesia using the combination of Zoletil (tiletamine-zolazepam, Virbac, Carros, France) and Rompun (xylazine-hydrochloride, Bayer, Leverkusen, Germany) (1:1, v/v). Blood sampling was performed from the ventral aorta and rapidly transferred into a BD Vacutainer (Franklin Lakes, NJ, USA). After 2 h of clotting, serum was separated by centrifuging the blood at 3,000 × g for 15 min at room temperature. Liver, pancreas, and fat tissues were removed quickly, washed and weighed, and stored in liquid nitrogen for future analysis.

S2 Cell culture

INS-1 (rat insulinoma cells), C2C12 (mouse myoblasts), and HepG2 (human hepatocarcinoma cells) (ATCC, Manassas, VA, USA) were used for the *in vitro* studies. They were cultured in RPMI-1640 (Gibco by Life Technologies, Carlsbad, CA, USA) or DMEM (Gibco by Life Technologies, Carlsbad, CA, USA) supplemented with different constituents as mentioned below. All cultured cell lines were maintained in an incubator at 37˚C containing humidified air of 5% CO2 at passages less than 25.

S3 Determination of insulin secretion and sensitivity, and glucose uptake in vitro

INS-1 cells were seeded at 2.5 × 10^5 cells per well into a 12-well plate (ThermoFisher Scientific, Waltham, MA USA) and cultured in RPMI-1640 (Gibco by Life Technologies, Carlsbad, CA, USA) containing 10% FBS (Gibco by Life Technologies, Carlsbad, CA, USA), 11 mM glucose, 1% penicillin/streptomycin, 10 mM HEPES, 1 mM sodium pyruvate, and 50 μM 2-mercaptoethanol. After 72 h, cells were treated for 48h with metformin (750 μM) either alone or in combination with HCE (100 and 200 μg/mL) in KRBB-HEPES medium (134 mM NaCl, 4.8 mM KCl, 1 mM CaCl2, 1.2 mM $MgSO_4$, 1.2 mM KH2PO_4, 5 mM $NaHCO_3$, 10 mM HEPES, 1 mg/ml BSA, pH 7.4) supplemented with 11 mM glucose. Following this, the medium was discarded and replaced with high glucose (20 mM)-containing KRBB-HEPES medium and the cells were incubated in this condition for 1.5 h. Insulin concentration of the cell supernatant was quantified using a Mercodia ELISA Rat Insulin Kit (Mercodia, Uppsala, Sweden) according to the kit manufacturer's protocol. Immediately, the cells were lyzed in lysis buffer (1% Triton-X, 20 mM HEPES, pH 7.4, 100 mM KCl, 2 mM EDTA, 1.0 mM PMSF, with protease inhibitors (Roche, Mannheim, Germany). Total protein of the cell lysate was measured using a BCA assay kit (Thermo Scientific, Rockford, IL, USA). The level of secreted insulin was normalized to the total cellular protein content.

C2C12 myocytes and HepG2 cells were seeded at 1 × 10^4 cells per well into 96-well black, clear bottom plates (Greiner Bio-One, Frickenhausen, Germany) and cultured for 24 h in glucose-

containing DMEM supplemented with 10% FBS. In case of C2C12 cells, the media was then discarded and replaced with glucose-containing DMEM supplemented with 2% horse serum. The C2C12 cells were incubated in this condition until differentiation of more than 90% of the cells was achieved (approximately 96 h). After 2 h wash out by FBS-free and glucose-free DMEM, HepG2 and differentiated C2C12 cells were treated with 10 mM glucosamine (Sigma-Aldrich, St. Louis, MO, USA) and/or 200 nM insulin (Eli Lilly and Company, Indianapolis, IN, USA) for 3 h. Finally, C2C12 and HepG2 cells were exposed to metformin (750 μM) either alone or in combination with HCE (50, 100 and 200 μg/mL) for 12 h followed by treatment with 75 μg/mL 2-deoxy-2-[(7-nitro-2,1,3-benzoxadiazol-4-yl)amino]-D-glucose (2-NBDG, Life Technologies, San Francisco, CA, USA) for 2 h. In glucose uptake assay, 50 μM apigenin was used as a negative control for comparison purpose. The uptake of 2-NBDG by the cells was detected by fluorescence microscopy (Olympus BX-61, Tokyo, Japan) and determined using a SpectraMax M3 fluorescence reader (Molecular Devices, Sunnyvale, CA, USA) with excitation and emission wavelengths at 475 and 515 nm, respectively.

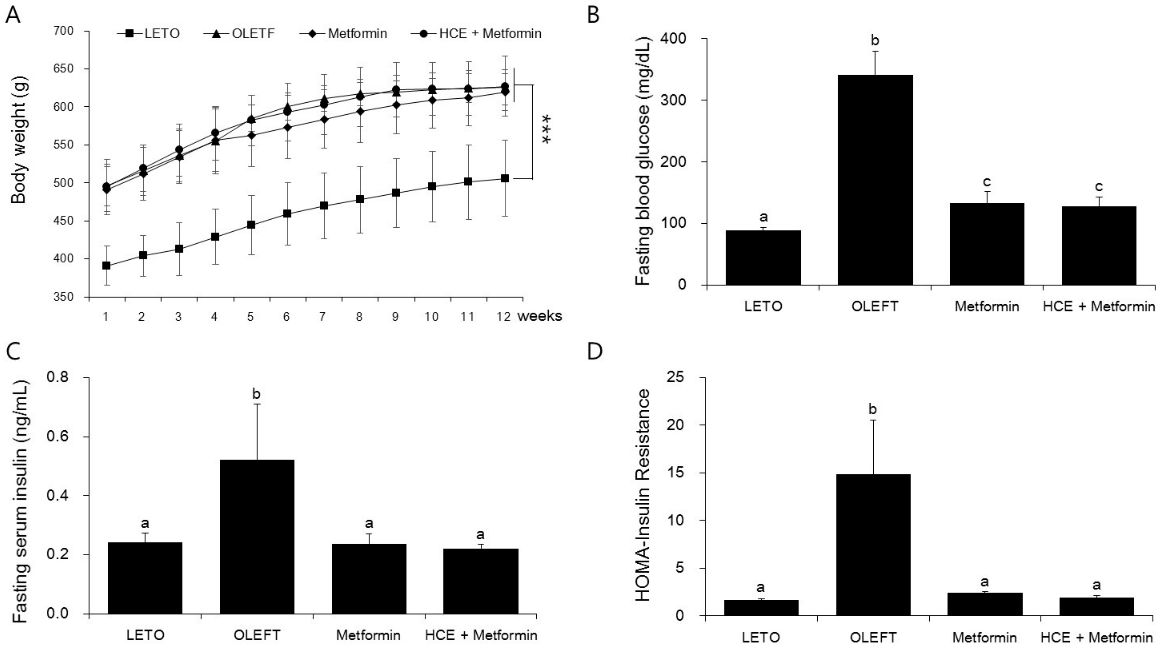

Supplementary Figure S1. Profile of body mass and glucose metabolism. Body weight of rats were recorded once per week (A). Fasting blood glucose (B) and serum insulin (C) were determined at the end of experimental schedule. (D) HOMA-insulin resistance was calculated as: [fasting blood insulin level (mg/dL) X fasting blood glucose level (ng/mL)]/405.

Supplementary Table S1. Body weight, fat and organ weights, food intake and food efficiency ratio, and other parameters of the different experimental groups

Groups	LETO	OLETF	Metformin	Metfomrin+HCE
Initial body weight (g)	392.1±27.7[a]	495.6±37.3[b]	491.6±35.6[b]	495.8±27.9[b]
Final body weight (g)	512.9±27.3[a]	612.9±23.7[b]	612.7±26.2[b]	616.6±42.5[b]
Food intake (g/week)	161.4±10.9[a]	226.1±9.8[b]	217.7±14.2[b]	217.2±18.0[b]
Food efficiency ratio (%)	11.5±0.4[a]	9.4±0.8[b]	9.8±0.7[b]	10.0±0.8[b]
Total fat (g)	18.9±3.2[a]	48.3±4.8[b]	47.7±8.0[b]	46.6±8.0[b]
Relative total fat (%)	3.2±1.5[a]	7.9±0.6[b]	7.8±1.1[b]	7.5±0.9[b]
Abdominal fat (g)	3.3±1.0[a]	9.6±1.1[b]	10.3±1.5[b]	9.9±1.5[b]
Relative abdominal fat (%)	0.7±0.2[a]	1.6±0.1[b]	1.7±0.2[b]	1.6±0.2[b]
Perirenal fat (g)	6.6±1.3[a]	23.7±3.6[b]	22.5±3.5[b]	21.5±4.5[b]
Relative perirenal fat (%)	1.3±0.2[a]	3.8±0.4[b]	3.7±0.5[b]	3.5±0.6[b]
Epididymal fat (g)	8.7±1.6[a]	14.8±3.1[b]	14.9±3.5[b]	15.2±2.5[b]
Relative epididymal fat (%)	1.7±0.3[a]	2.4±0.6[b]	2.4±0.5[b]	2.4±0.3[b]
Kidney weight (g)	6.6±1.3[a]	23.7±3.6[b]	22.5±3.5[b]	21.5±4.5[b]
Relative kidney weight (%)	1.3±0.2[a]	3.8±0.4[b]	3.7±0.5[b]	3.5±0.6[b]
Liver weight (g)	12.8±0.9[a]	20.5±2.0[b]	18.3±2.9[b]	19.2±2.9[b]
Relative liver weight (%)	2.5±0.2[a]	3.2±0.3[b]	3.0±0.4[b]	3.1±0.3[b]

Abbreviations: Rela. of fat, relative weight of total fat; Relative tissues weight (%)=(tissue weight/body weight) x 100%. Food efficiency ratio = body weight gain (g/day) / food intake (g/day). Data were expressed as Mean ± SD, different letters indicate significantly different according to one way ANOVA analysis followed by LSD post-hoc test ($P < 0.05$, n = 7).

Supplementary Table S2. List of primers used for the gut microbial analysis by real-time PCR

Bacteria	Primer sequence	OAT
Gram negative bacterium	5'-AYG ACG TCA AGT CMT CAT GG-3'<br>5'-AAC TGG AGG AAG GTG GGG AY-3'	65°C
Prevotella spp.	5'-CCA GCC AAG TAG CGT GCA-3'<br>5'-TGG ACC TTC CGT ATT ACC GC-3'	65°C
Roseburia spp.	5'-GCG GTR CGG CAA GTC TGA-3'<br>5'-CCT CCG ACA CTC TAG TMC GAC-3'	60°C
Akkermansia spp.	5'-CAG CAC GTG AAG GTG GGG AC-3'<br>5'-CCT TGC GGT TGG CTT CAG AT-3'	60°C
Faecalibacterium prausnitzii	5'-GGA GGA AGA AGG TCT TCG G-3'<br>5'-AAT TCC GCC TAC CTC TGC ACT-3'	60°C
Echerichia coli	5'-GAC CCG GCA CAA GCA TAA GC-3'<br>5'-CCA CCT GCA GCA ACA AGA GG-3'	65°C
Lactobacillus brevis	5'-CTT GCA CTG ATT TTA ACA-3'<br>5'-GGG CGG TGT GTA CAA GGC-3'	55°C
Lactobacillus acidophillus	5'-AGC TGA ACC AAC AGA TTC AC-3'<br>5'-ACT ACC AGG GTA TCT AAT CC-3'	64°C

Abbreviations: OAT, optimized annealing temperature

Supplementary Table S3. List of antibodies used for western blot analysis

Peptide/protei n target	Manufacturer, catalog #, and/or name of individual providing the antibody	Species raised in; monoclonal or polyclonal	Dilution used
Beta-actin	Santa Cruz, #sc-47778	Mouse monoclonal	1:2000
AMPK	Cell Signaling, #2532	Rabbit polyclonal	1:1000
Phosphorylated AMPK	Cell Signaling, #2535	Rabbit polyclonal	1:1000
